# Kupffer cell receptor CLEC4F is important for the destruction of desialylated platelets in mice

**DOI:** 10.1038/s41418-021-00797-w

**Published:** 2021-05-15

**Authors:** Yizhi Jiang, Yaqiong Tang, Christopher Hoover, Yuji Kondo, Dongping Huang, Damien Restagno, Bojing Shao, Liang Gao, J. Michael McDaniel, Meixiang Zhou, Robert Silasi-Mansat, Samuel McGee, Miao Jiang, Xia Bai, Florea Lupu, Changgeng Ruan, Jamey D. Marth, Depei Wu, Yue Han, Lijun Xia

**Affiliations:** 1 Jiangsu Institute of Hematology, National Clinical Research Center for Hematologic Diseases, NHC Key Laboratory of Thrombosis and Hemostasis, The First Affiliated Hospital of Soochow University, Suzhou, 215006 China; 2 Department of Hematology, The First Affiliated Hospital of Wannan Medical College, Wuhu, 241001 China; 3 Cardiovascular Biology Research Program, Oklahoma Medical Research Foundation, Oklahoma City, OK 73104 USA; 4 Collaborative Innovation Center of Hematology, Soochow University, Suzhou, 215006 China; 5 State Key Laboratory of Radiation Medicine and Protection, Soochow University, Suzhou, 215123 China; 6 Center for Nanomedicine, SBP Medical Discovery Institute, and Department of Molecular, Cellular, and Developmental Biology, University of California, Santa Barbara, CA 93106 USA

**Keywords:** Glycobiology, Cell death and immune response, Haematological diseases

## Abstract

The liver has recently been identified as a major organ for destruction of desialylated platelets. However, the underlying mechanism remains unclear. Kupffer cells, which are professional phagocytic cells in the liver, comprise the largest population of resident tissue macrophages in the body. Kupffer cells express a C-type lectin receptor, CLEC4F, that recognizes desialylated glycans with an unclear in vivo role in mediating platelet destruction. In this study, we generated a CLEC4F-deficient mouse model (*Clec4f*^−/−^) and found that CLEC4F was specifically expressed by Kupffer cells. Using the *Clec4f*^−/−^ mice and a newly generated platelet-specific reporter mouse line, we revealed a critical role for CLEC4F on Kupffer cells in mediating destruction of desialylated platelets in the liver in vivo. Platelet clearance experiments and ultrastructural analysis revealed that desialylated platelets were phagocytized predominantly by Kupffer cells in a CLEC4F-dependent manner in mice. Collectively, these findings identify CLEC4F as a Kupffer cell receptor important for the destruction of desialylated platelets induced by bacteria-derived neuraminidases, which provide new insights into the pathogenesis of thrombocytopenia in disease conditions such as sepsis.

## Introduction

Platelets are abundant, yet short-lived, blood cells whose number is second only to red blood cells within circulation. Platelet function is multifaceted, comprising important physiological processes such as hemostasis, vascular integrity, and immunity [1–6]. The lifespan of human platelets is 8–10 days, while in mice it is only 4–5 days. The number of platelets in the blood is controlled through the balance of their rate of production in the bone marrow and lung with their clearance in peripheral compartments that can include the spleen and liver. To maintain a stable platelet count, approximately 10^11^ human platelets are produced and removed daily from circulation while the dysregulation of this homeostatic process can contribute to multiple pathological conditions such as sepsis and immune thrombocytopenia [4, 7–10].

There have been many critical advances made in the understanding of platelet biogenesis, but the molecular mechanisms underlying platelet clearance from circulation have remained, in part, mysterious [11]. Fc–FcγR-dependent phagocytosis via macrophages in the spleen, T-cell-mediated destruction, and platelet apoptosis have been identified as participating in platelet clearance [8, 11, 12]. However, those processes appear insufficient to account for all platelet clearance [4, 7]. An alternative turnover system is predicted.

Most platelet membrane receptors are glycoproteins (GP), including the GPIb-IX–V complex and integrin αIIbβ3 (GPIIb/IIIa). Major forms of glycosylation include *N*-linked glycans (*N*-glycans) and mucin-type *O*-linked glycans (*O*-glycans), which are commonly “capped” by sialic acids, an enzymatic process termed sialylation [9, 11]. α2,3-linked sialic acid is the main form of platelet sialylation, in which sialic acid is commonly linked to the galactose (Gal) or N-acetylgalactosamine (GalNAc) on *N*-glycans and *O*-glycans. The sialylation level on platelets is reduced in conditions such as cold storage of platelets, sepsis, and immune thrombocytopenia [3, 4, 8, 11, 13]. Neuraminidases (sialidases), which exist in many pathogens such as viruses and bacteria, as well as in mammalian cells, are glycosidase enzymes that catalyze the hydrolysis of sialic acid linkages and removal of sialic acids [14].

Liver is the largest solid organ. Hepatocytes have been proposed to mediate the clearance of desialylated platelets. The asialoglycoprotein receptor (or Ashwell Morell receptor, AMR), a C-type lectin receptor that binds glycans including those bearing exposed β-gal or β-GalNAc, has been reported to mediate the phagocytosis of desialylated platelets by hepatocytes [3, 4, 7, 15, 16]. However, the conclusion was primarily based on phagocytosis of desialylated or cold-stored platelets by a hepatocyte cell line (hepG2 cells) in vitro. It is unclear in vivo whether platelets would come in contact with hepatocytes that are separated from the hepatic sinusoidal microcirculation by the sinusoidal endothelium and the perisinusoidal space (space of Disse) [17]. The size of liver sinusoidal endothelial fenestrates (~180 nM in size) is much smaller than that of platelets (~500 nm in diameter) so that platelets cannot pass through readily [9]. The liver also contains an abundance of a professional type of phagocytes known as Kupffer cells, which comprises the largest population of resident tissue macrophages in the body [17, 18]. Our recent study supports Kupffer cells, instead of hepatocytes, in playing the major role in clearance of desialylated platelets [9].

Kupffer cells express multiple endocytic lectin receptors including a C-type lectin CLEC4F (Kupffer cell receptor) [19, 20]. CLEC4F has high binding affinity for desialylated glycans bearing exposed β-gal or β-GalNAc. CLEC4F was first purified from rat liver extract and exhibits a higher affinity for desialylated glycan moieties with a Gal, GalNAc, or fucose terminus [9, 21–23]. However, the expression pattern and biological function of CLEC4F, in regards to the destruction of desialylated platelets in vivo, has remained unclear. In this study, we generated new CLEC4F-deficient mouse lines (*Clec4f*^−/−^) and demonstrated an important role for CLEC4F on Kupffer cells in mediating the clearance of desialylated platelets induced by bacteria-derived neuraminidases in the liver in vivo.

## Results

### CLEC4F is specifically expressed by Kupffer cells

To determine the expression pattern and function of CLEC4F, we generated *Clec4f*^-−/−^ mice by CRISPR/Cas9-mediated gene targeting (Supplementary Fig. S1a–d). The gRNA targeting the *Clec4f* exon 2 and saCas9 mRNA were injected into C57BL/6J zygotes, which were then transferred into the oviduct of CD-1 foster females. Direct sequencing of PCR products using tail-derived genomic DNA identified bi-allelic frameshifting indel-mutations of *Clec4f* from a founder. Two independent F1 lines with two different heritable indel-mutations, termed *Clec4f* −4 and *Clec4f* −6 + 1, were then generated by crossing the founder with a wild-type (WT) C57BL/6J mouse. Both F1 heterozygous lines were further bred to generate homozygous *Clec4f* −4 and *Clec4f* −6 + 1 lines (Supplementary Fig. S1e, f). To facilitate the study, we primarily used the *Clec4f* −4 mouse line (hereafter *Clec4f*^-−/−^ unless specified) as a model. In some experiments, the *Clec4f* −6 + 1 line was used to validate the results.

**Fig. 1:**
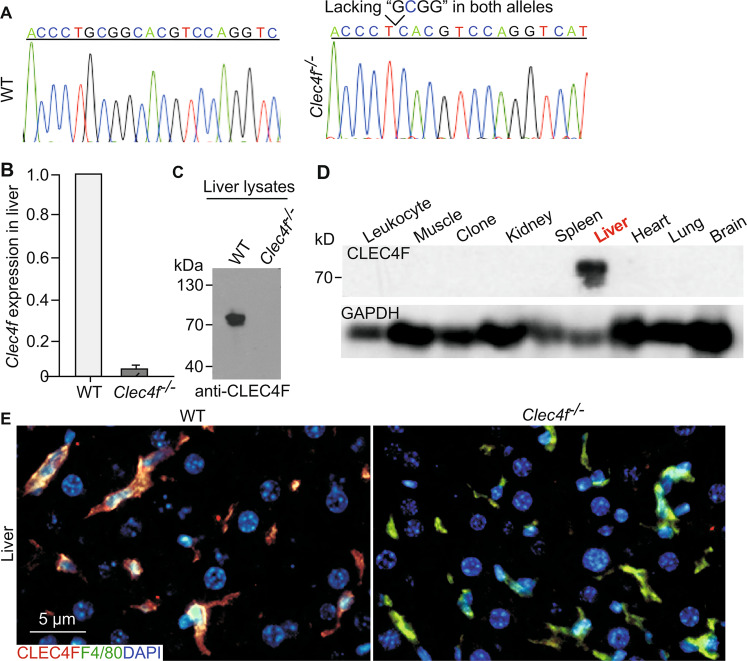
CLEC4F is specifically expressed by Kupffer cells in the mouse liver. **A** The sequencing results of *Clec4f*^−/−^ mice with deletion of 4-bp “GCGG” in both *Clec4f* alleles. **B** *Clec4f* mRNA expression in the mouse liver analyzed by qRT-PCR. **C** CLEC4F protein levels of mouse liver lysates analyzed by western blot. **D** CLEC4F protein levels of different mouse tissue lysates analyzed by western blot**. E** Representative images of immunofluorescent staining of formalin-fixed paraffin-embedded mouse liver sections. Kupffer cell (CLEC4F, red or F4/80, green). Results are representative of at least three experiments.

Further DNA sequencing results confirmed that *Clec4f*^−/−^ mice exhibited a 4-bp (GCGG) frameshifting deletion in *Clec4f* exon 2 (Fig. 1A). Quantitative RT-PCR and western blot analyses indicated that the homozygote deletions resulted in a loss of *Clec4f* mRNA transcripts as well as CLEC4F protein relative to that of WT littermate controls (Fig. 1B, C). *Clec4f*^−/−^ mice of both sexes appeared healthy with a normal life-span. Compared with WT mice, *Clec4f*^−/−^ mice showed normal peripheral blood cell counts (Supplementary Fig. S2a). Similar to findings reported in AMR deficiency [4], the number of peripheral platelets in *Clec4f*^−/−^ mice was comparable to that of WT littermates. Flow cytometry analyses indicated that *Clec4f*^−/−^ peripheral platelets had no significant difference in their sialylation status and responses to agonists such as thrombin compared with WT platelets (Supplementary Fig. S2b). These data indicated that lack of CLEC4F does not alter platelet homeostasis under normal physiological conditions.

To examine the CLEC4F expression pattern, we performed western blot analysis and immunofluorescent staining of formalin-fixed paraffin-embedded tissue sections. Western blot analysis of multiple WT mouse organs showed that CLEC4F was only detected in the liver (Fig. 1D). Confocal microscopy imaging results indicated that CLEC4F was specifically colocalized with F4/80 positive Kupffer cells in WT but not *Clec4f*^−/−^ mouse liver sections (Fig. 1E). Moreover, CLEC4F was not detectable in hepatocytes (Fig. 1E) or the spleen of either WT or *Clec4f*^−/−^ mice (Supplementary Fig. S2c). These data indicate that CLEC4F is specifically expressed by the Kupffer cell in the mouse liver and validate that our new mutant mouse lines lack CLEC4F protein.

To explore the potential human biology relevance of CLEC4F, we examined a de-identified formalin-fixed paraffin-embedded human autopsy liver sample for CLEC4F expression. Our immunostaining detected CLEC4F in the human liver sample (Supplementary Fig. S3a). In addition, we found that most CLEC4F staining colocalized with CD68-positive Kupffer cells in the human liver sample (Supplementary Fig. S3a).

### Kupffer cell CLEC4F mediates the rapid clearance of platelets desialylated in vivo by bacteria-derived neuraminidases

**Fig. 2:**
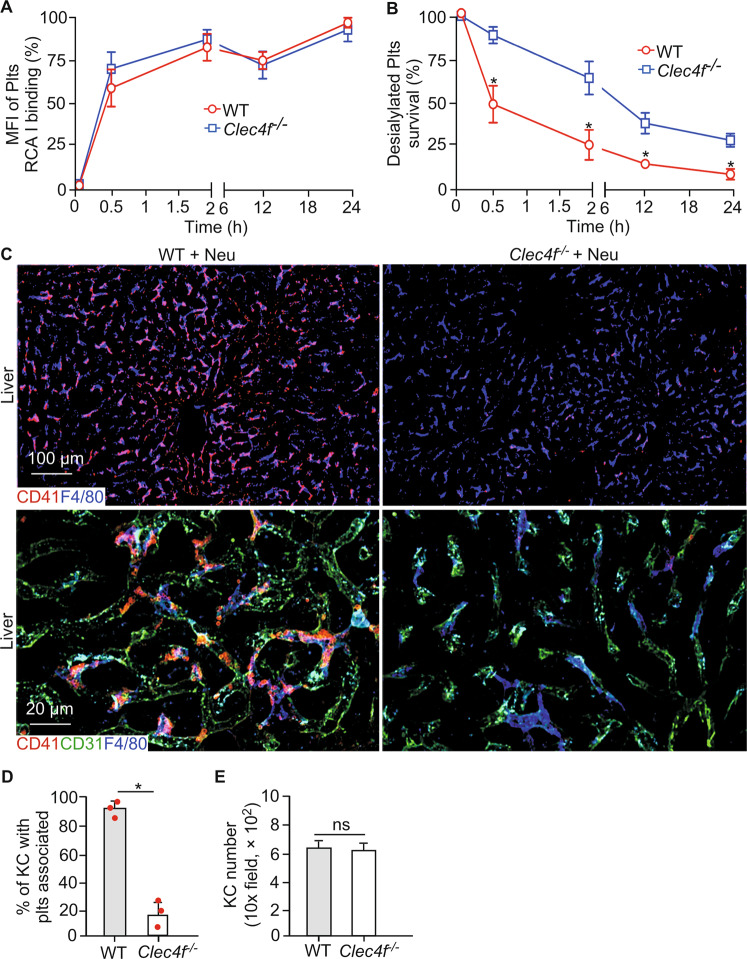
Kupffer cell CLEC4F is a dominant receptor in mediating the rapid clearance of desialylated platelets in the liver. **A** Flow cytometry analysis of galactose exposure (RCA I binding) on platelets of either WT or *Clec4f*^−/−^ recipients treated with *Arthrobacter ureafaciens*-derived neuraminidase at different time points. MFI, Mean fluorescence intensity. **B** Percentage of platelets of either WT or *Clec4f*^−/−^ recipients treated with *Arthrobacter ureafaciens*-derived neuraminidase at different time points. Platelet count was measured using flow cytometric analysis of peripheral blood based on anti-CD41. **C** Representative immunofluorescent images showing association of platelets (CD41, red) with Kupffer cells (F4/80, blue) in the liver sections. Endothelium, (CD31, green). **D**, **E** Quantification of association of platelets with liver Kupffer cells or Kupffer cell (KC) number at 2 h after *α* neuraminidase injection. *n* = 5–10 images/10 × fields/experiment. Data represent means ± SEM from three experiments. *P < 0.05.

Thrombocytopenia is commonly associated with severe infections such as sepsis. Pathogen-derived neuraminidases are a cause of platelet desialylation and clearance in some severe infections [3, 4]. We detected a marked increase in the percentage of platelet RCA1 binding of both WT and *Clec4f*^−/−^ mice within the initial 30 min after injection of *Arthrobacter ureafaciens*-derived neuraminidase (Fig. 2A), which was continuously increased at 24 h post-neuraminidase administration. We found that WT mice exhibited a faster clearance rate of endogenous platelets than *Clec4f*^−/−^ mice, with a major reduction of platelets within the initial 30 min (Fig. 2B) following the administration of the neuraminidase. However, there was no obvious difference in the clearance of other types of blood cells between WT and *Clec4f*^−/−^ mice following neuraminidase treatment (Supplementary Fig. S3b).

**Fig. 3:**
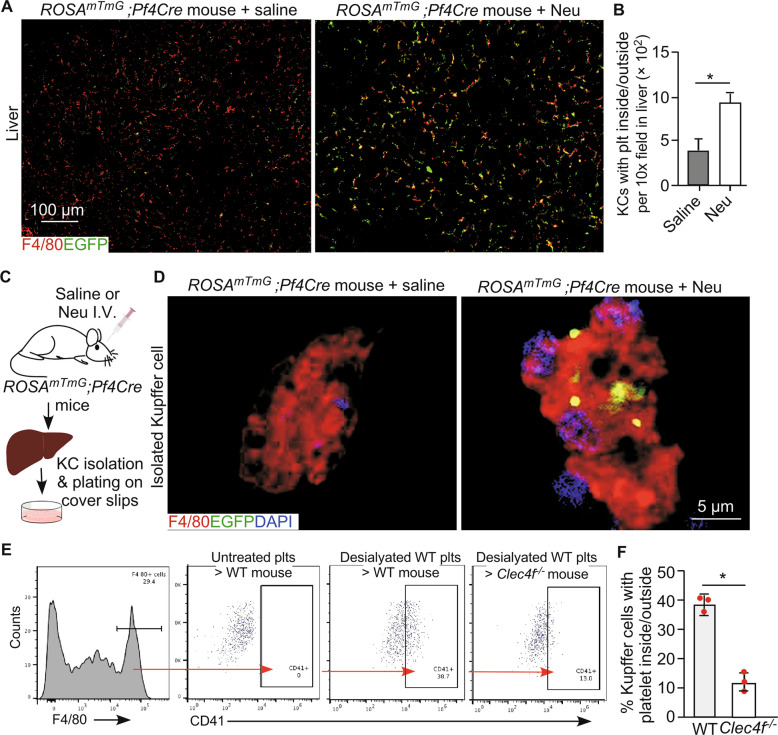
Kupffer cell CLEC4F regulates phagocytosing of desialylated platelets in vivo. **A** Confocal microscopic images showing association of platelets (EGFP, green) with Kupffer cells (F4/80, red) in the liver 2 h after neuraminidase treatment. **B** Quantification of EGFP-positive platelets associated with Kupffer cells. Data represent means ± SD. *n* = 5–10 images/10 × fields. *P < 0.05. **C** Schematic diagram for isolation of Kupffer cells from *ROSA*^*mTmG*^;*Pf4Cre* mouse 2 h after neuraminidase treatment. **D** Representative high-resolution confocal microscopic images of an individual Kupffer cell (F4/80), isolated from *ROSA*^*mTmG*^*;Pf4Cre* mice 2 h after neuraminidase treatment, with platelets presented inside (EGFP, green, right panel). Control, saline treated. Neu, neuraminidase. I.V., intravenous injection. **E** Flow cytometry analysis of liver Kupffer cells isolated from mice transfused with platelets with or without desialylation as indicated on the top of each panel. Left panel shows the F4/80 positive Kupffer cell gating for analysis. Right panels (red arrow) show results of platelets adherent to Kupffer cells from WT or *Clec4f*^−/−^ recipients transfused with control or desialylated platelets. **F** Quantification of the flow cytometry results. Data represent means ± SD, *n* = 3. *P < 0.05.

To determine the localization of the endogenous desialylated platelets, we performed confocal imaging analysis of liver sections from WT and *Clec4f*^−/−^ mice 2 h after neuraminidase administration. There was a significant increase in numbers of platelets associated with Kupffer cells in the WT than those in the in the *Clec4f*^−/−^ mouse liver (Fig. 2C, D). No obvious association of platelets with either hepatocytes or the sinusoidal endothelium was detected in the WT and *Clec4f*^−/−^ mouse liver (Fig. 2C, lower panels). Furthermore, there were no detectable differences for platelets in the spleen between WT and *Clec4f*^−/−^ mice (Supplementary Fig. S4). The number of Kupffer cells was comparable between WT and *Clec4f*^−/−^ mice (Fig. 3E).

### CLEC4F expressing Kupffer cells phagocytize desialylated platelets in the liver

To provide in vivo evidence that the CLEC4F-mediated interaction of desialylated platelets with Kupffer cells leads to phagocytosis, we generated a new megakaryocyte-/platelet-specific reporter mouse line by crossing the dual fluorescent reporter *ROSA*^*mTmG*^ mice with a transgenic mouse line expressing Cre recombinase under the control of the *Pf4* promoter. This *ROSA*^*mTmG*^*;Pf4Cre* reporter mouse line specifically expresses enhanced green fluorescent protein (EGFP) in megakaryocytes and platelets (Fig. 3A, D; Supplementary Fig. S5a), and tdTomato fluorescence in the membrane of all other cell types (Supplementary Fig. S5b). We found that EGFP-positive desialylated platelets were primarily associated with Kupffer cells in the liver (Fig. 3A, B), but not in other organs (Supplementary Fig. S5b), 2 h after intravenous injections of *Arthrobacter ureafaciens*-derived neuraminidase. In addition, we performed confocal microscopy imaging of individual Kupffer cells freshly isolated from mice following neuraminidase treatment and found that desialylated platelets were primarily present inside of each Kupffer cell (Fig. 3C, D). These results support that desialylated platelets are phagocytized by Kupffer cells in vivo.

To obtain further evidence that Kupffer cell CLEC4F contributes to the phagocytosis of desialylated platelets, we transfused isolated EGFP-positive platelets from the *ROSA*^*mTmG*^*;Pf4Cre* mice into either WT or *Clec4f*^−/−^ recipient mice. Platelets were treated by neuraminidase after isolation. Two h after transfusion, F4/80-positive Kupffer cells were isolated from the recipient mice and analyzed by flow cytometry. The endogenous EGFP signal was initially used to detect the transfused platelets. However, we found the EGFP signal was not strong enough to be detected by flow cytometry after in vitro treatment. Therefore, anti-CD41 staining of isolated non-permeabilized Kupffer cells was used in subsequent experiments, which detected primarily desialylated platelets bound to the Kupffer cell surface. Our results indicated that desialylated platelets specifically interacted with CLEC4F-expressing but not CLEC4F-deficient Kupffer cells (Fig. 3E, F).

**Fig. 4:**
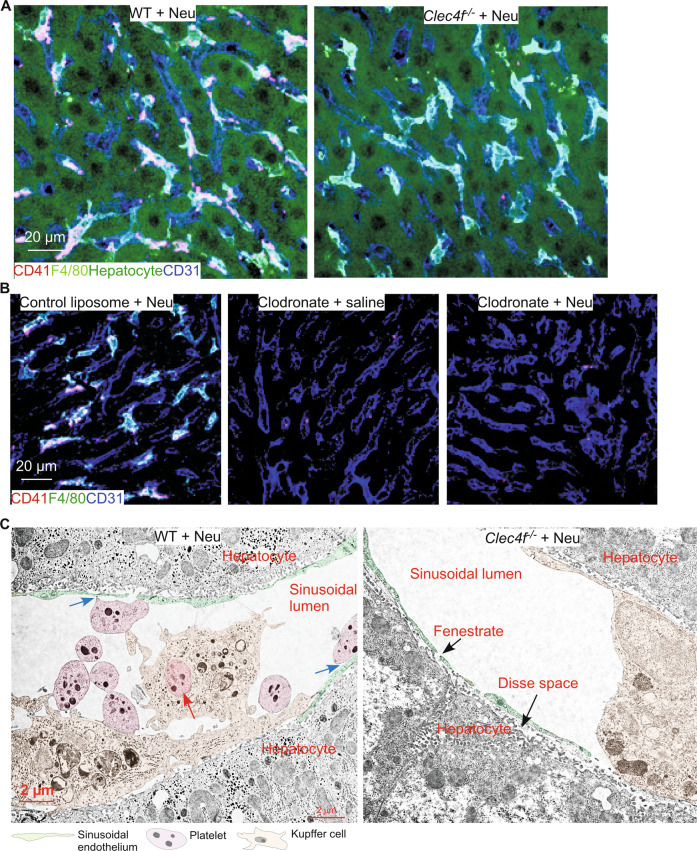
Kupffer cells are the primary cell type phagocytizing desialylated platelets in the liver. **A**, **B** Representative confocal microscopic images. CD41 marks platelets, F4/80 marks Kupffer cells, CD31 labels sinusoidal endothelium, and hepatocytes were detected by autofluorescence. **C** Pseudocolored TEM micrographs of WT or *Clec4f*^−/−^ ultrathin sections. Red arrow marks a phagocytized platelet. Blue arrows mark potential interactions between platelets and sinusoidal endothelium.

Previous studies report that hepatocytes phagocytize desialylated platelets [7, 24]. To test this, we immunostained liver cryosections of WT and *Clec4f*^*−/−*^ mice 2 h after neuraminidase treatment. Our confocal imaging results indicated that desialylated platelets were only associated with Kupffer cells but not with hepatocytes and sinusoidal endothelial cells (Fig. 4A). In addition, depletion of Kupffer cells using clodronate treatment abolished clearance of desialylated platelets in the WT liver after neuraminidase treatment (Fig. 4B), indicating the critical role of Kupffer cells in the clearance of desialylated platelets.

To provide definitive evidence demonstrating the phagocytosis of desialylated platelets by Kupffer cells, we performed TEM imaging of ultrathin liver sections of WT and *Clec4f*^*−/−*^ mice after neuraminidase treatment. TEM micrographs clearly demonstrated interactions of desialylated platelets with WT Kupffer cells (Fig. 4C). Importantly, desialylated platelets were readily detected inside WT Kupffer cells, demonstrating phagocytosis (Fig. 4C). In contrast, no desialylated platelets were found to interact with *Clec4f*^*−/−*^ Kupffer cells (Fig. 4C). Interestingly, some desialylated platelets appeared to interact with sinusoidal endothelial cells in the WT liver. Nevertheless, no desialylated platelets were detected within or associated with hepatocytes after extensive search of numerous TEM grids. These results demonstrate that it is the Kupffer cell that phagocytizes desialylated platelets in the liver, at least in the conditions we tested. In addition, these results also indicate that the Kupffer cell CLEC4F receptor is important for phagocytizing desialylated platelets.

### CLEC4F is an important receptor for phagocytosing of desialylated platelets by the Kupffer cell in vivo

To determine whether CLEC4F is a major receptor for mediating the phagocytosis of desialylated platelets in the liver, we first validated our results using a different type of bacteria-derived neuraminidase (*Clostridium perfringens*) that was used in previous related studies [4, 25]. Two independent CLEC4F-deficient mouse lines and mice with AMR deficiency (*Asgr1*^*−/−*^), which were used in previous studies [25], were used in this experiment. Injections of *Clostridium perfringens*-derived neuraminidase caused desialylation of platelets in these models measured by increased binding to RCA1 (Supplementary Fig. S6a), similar to results with *Arthrobacter ureafaciens*-derived neuraminidase (Fig. 2A). Also similar to results with *Arthrobacter ureafaciens*-derived neuraminidase (Fig. 2B), thrombocytopenia occurred within 2 h after neuraminidase injections. Confocal microscopy imaging of immunostained liver cryosections indicated that there was marked increase in numbers of platelets co-localized with Kupffer cells in WT liver 2 h after injections of neuraminidase. In contrast, there was dramatic decrease in the number of platelets in the liver of two different lines of CLEC-deficiency after neuraminidase treatment (Supplementary Fig. S6b). As previously reported, there was a slight decrease in numbers of platelets in association with Kupffer cells in the liver of *Asgr1*^*−/−*^ mice. Immunostaining indicated that AMR expression was comparable between WT and different *Clec4f*^−/−^ mouse livers (Supplementary Fig. S7), suggesting that deficiency of CLEC4F did not unexpectedly alter the expression of AMR.

**Fig. 5:**
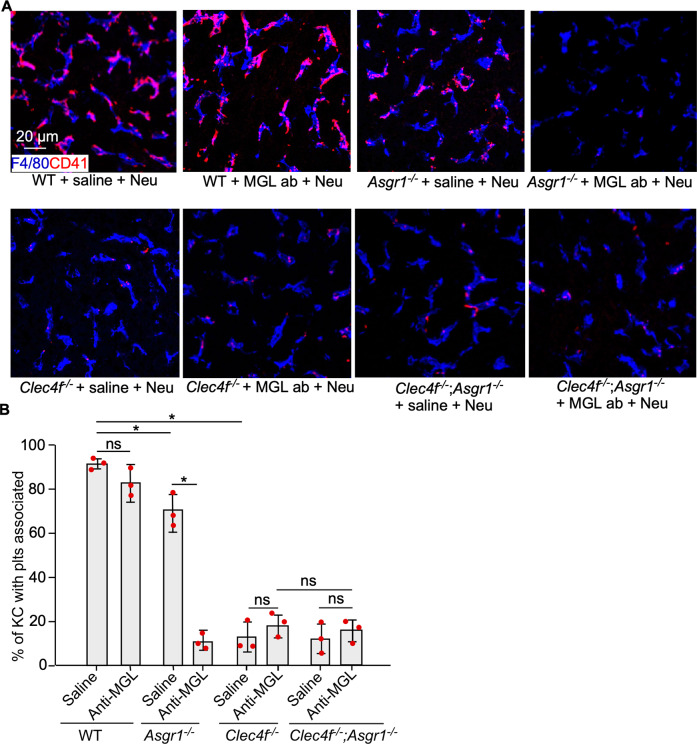
CLEC4F is critical to the Kupffer cell-mediated phagocytosis of desialylated platelets. **A** Representative confocal microscopic images of desialylated platelets (CD41, red) and Kupffer cells (F4/80, blue) in liver sections of different mouse models. **B** Quantification of platelets with liver Kupffer cells of different mouse models 2 h after *Clostridium perfringens*-derived neuraminidase injection. *n* = 5–10 images/20 × fields/mouse. Data represent means ± SEM from three experiments. *P < 0.05. ns, not significant.

Other than AMR and CLEC4F, the MGL on Kupffer cells is also considered important for the clearance of desialylated platelets [4, 7, 25]. However, the differential contributions of these receptors to the clearance of desialylated platelets have been unclear. Therefore, we directly compared WT mice and mice with either genetically lacking or antibody-mediated functional blocking of MGL, AMR, or CLEC4F, individually or in combination, after in vivo desialylation based on procedures described above. Consistent with previous studies [4, 25], deficiency of AMR or MGL alone did not significantly reduce the clearance of desialylated platelets in the liver (Fig. 5A). However, lack of CLEC4F or both AMR and MGL significantly reduced the association of desialylated platelets with Kupffer cells (Fig. 5A, B). Loss of AMR or MGL did not further reduce desialylated platelet clearance in the *Clec4f*^−/−^ liver.

VWF and GPIbα are reported to mediate platelet interactions with Kupffer cells during inflammation [26]. In addition, desialylation may activate platelets and leukocytes to form platelet/leukocyte complexes mediated by platelet P-selectin and its ligand PSGL-1 on leukocytes [27, 28], which may affect the clearance of desialyated platelets. We, therefore, examined these possibilities using *VWF*^*−/−*^ mice and mice treated with RB40 (blocking P-selectin function) or 4RA10 (a functional blocker to PSGL-1, both antibodies were from Dr. Dietmar Vestweber, Germany). Our results showed blockage of VWF or P-selectin pathway did not affect the clearance of desialylated platelet by the Kupffer cell (Supplementary Fig. S8).

Collectively, these data demonstrate that CLEC4F is important to the Kupffer cell-mediated phagocytosis of desialylated platelets induced by bacteria-derived neuraminidases.

## Discussion

Recent studies have shown the liver to be the major site for the clearance of desialylated platelets [4, 7, 9, 24, 25, 29]. However, the cell type and receptor in the liver that regulate the clearance of desialylated platelets in vivo remain controversial [9]. Using newly generated *Clec4f*^−/−^ mice and a platelet-specific reporter mouse line, our results revealed that desialylated platelets primarily bind to CLEC4F specifically on Kupffer cells in the mouse liver and that Kupffer cells are the predominant cell phagocytozing platelets desialylated by bacteria-derived neuraminidases in vivo.

Since 2008, studies have shown both that platelet desialylation is critical for platelet clearance in many pathological states and the liver is the major site for the clearance of desialylated platelets [4]. However, which cell type in the liver phagocytizing desialylated platelets in vivo has remained elusive. Several previous studies show that hepatocytes phagocytize desialylated platelets, which is mediated by the AMR [4, 24]. In contrast, recent studies from our group and others have demonstrated that Kupffer cells are the primary cells that bind desialylated platelets [9, 25]. Kupffer cells are a professional type of phagocyte in the liver and uniquely positioned within the hepatic sinusoids, which allows them to directly interact with and phagocytize desialylated platelets entering from the portal and/or arterial circulation. Our current study indicates that desialylated platelets are predominantly phagocytized by Kupffer cells. We found that the clearance of desialylated platelets in the liver was significantly reduced after removal of Kupffer cells by clodronate liposomes. Importantly, TEM imaging demonstrates, for the first time to our knowledge, phagocytized platelets in Kupffer cells. However, our TEM imaging did not detect any platelets inside or associated with hepatocytes of mice treated with neuraminidase. Taken together, these data provide definitive evidence indicating that the Kupffer cell is the primary cell phagocytizing desialylated platelets, at least in the conditions we tested.

Several previous studies have reported that the hepatocyte AMR participates in the clearance of desialylated platelets [3, 4, 7]. A recent study reported that both the AMR and the macrophage galactose lectin (MGL), which is expressed on the Kupffer cell, are required for Kupffer cells to bind desialylated platelets induced by *Clostridium perfringens*-derived neuraminidase [25]. In this study, we directly compared the differential contributions of these receptors to the clearance of desialylated platelets. Our data show that loss of CLEC4F itself is sufficient to impair the clearance of desialylated platelets by the Kupffer cell in the liver. These seemingly discrepant results strongly suggest a complex and coordinated mechanism of regulating the clearance of desialylated platelets in the liver. These three receptors, AMR, MGL, and CLEC4F, are closely related lectins. Although AMR has been reported to be specifically expressed on hepatocytes, recent publications and our staining indicate that AMR may also be expressed at much lower levels on Kupffer cells [25] (Supplementary Fig. S7). In addition, MGL and CLEC4F are specifically expressed on Kupffer cells in the liver of mice [30]. Considering our data and recent published findings [9], the adhesion and phagocytosis of desialylated platelets mediated by these lectins in the hepatic sinusoidal microcirculation may be reminiscent of the well-defined P- and E-selectin-mediated leukocyte recruitment paradigm in the inflamed postcapillary venules under flow conditions [28, 31]. It is tempting to speculate that the AMR and MGL may mediate an initial tethering and adhesion, and that the MGL and/or CLEC4F function to mediate firm adhesion and the phagocytosis of desialylated platelets. Indeed, our TEM images demonstrate that some desialylated platelets tether to and adhere on the sinusoidal endothelial cells, which might be mediated by AMR on the hepatocyte microvilli penetrating through sinusoidal endothelial fenestraes. Further studies are required to determine details regarding how these lectin receptors coordinate their actions to clear platelets in the liver.

Our data indicate that CLEC4F is specifically expressed in liver F4/80 positive Kupffer cells under physiological conditions, but not in other tissues, indicating that CLEC4F is a Kupffer cell-specific receptor and marker in mice. A recent study, primarily based on bioinformatics analysis, reported that humans do not have a functional CLEC4F receptor due to a mutation in the splice acceptor site of the final exon on the human CLEC4f gene that prevents appropriate splicing, as well as an additional missense mutation that disrupts the sugar-binding site [23]. However, our results showed that CLEC4F was detected in a human liver tissue sample and colocalized with a Kupffer cell marker CD68. This result suggests that humans may express a functional CLEC4F, although a systemic study of human CLEC4F for its expression pattern and function, which is beyond the scope of the current study, is required. In addition, the repertoire of CLEC4F functions in mice and how those functions may have been accommodated by the evolution by other endocytic lectins expressed on Kupffer cells in humans, such as possibly MGL [25], requires additional future studies. Nevertheless, mouse models are commonly used to study desialylation-dependent thrombocytopenia. Our results, therefore, are important for the interpretation of results in this regard.

In this study, our findings reveal CLEC4F as a novel Kupffer cell lectin receptor for platelets with β-gal or β-GalNAc glycans that are exposed in the presence of different bacterial neuraminidases. The relevance between increased platelet clearance in the liver and disorders with platelet desialylation, such as sepsis, has recently attracted significant research interest [3, 4, 7]. Neuraminidases are commonly found in many sepsis-causing pathogens such as bacteria [3]. Therefore, our study will have a unique and significant impact on the understanding of host coagulopathy and inflammation in sepsis caused by bacterial pathogens. Other than pathogen-derived neuraminidases, the mammalian cells also express neuraminidases. Autoantibodies against GPIbα or GPIIbIIIa in patients with immune thrombocytopenia (ITP) induce the secretion of endogenous neuraminidases from platelets, leading to Fc–FcγR-independent clearance of desialylated platelets in the liver [8, 13]. ITP patients with no response to standard first-line therapies had significantly higher levels of platelet desialylation compared to healthy controls and responders. Future studies in these pathological conditions will provide new insights into the role of Kupffer cell receptor CLEC4F in disorders with thrombocytopenia such as sepsis and ITP.

## Materials and methods

### Mice

*Clec4f*-deficient (*Clec4f*^−/−^) mice were generated using CRISPR/Cas9-mediated gene targeting. *ROSA*^*mTmG*^ mice (#007576, the Jackson Laboratory) were crossed with a transgenic mouse line expressing Cre recombinase under the control of the mouse *Pf4* promoter (*Pf4Cre* mice, #008525, the Jackson Laboratory) to generate megakaryocyte-/platelet-specific reporter mice (*ROSA*^*mTmG*^*;Pf4Cre*). Mice lacking von Willebrand factor (*VWF*^*−/−*^) were from Dr. David Ginsburg. Mice were kept in a specific-pathogen free facility. Mice genotypes were determined by using the tail-derived genomic DNA. All mice are on a C57Bl/6 background. Sex- and age-matched WT littermates were used for controls. Mice between 6 and 12 weeks of age were used unless otherwise stated. All experimental procedures were approved by the Institutional Animal Care and Use Committee of the Oklahoma Medical Research Foundation.

### Key antibodies and reagents

The following are primary reagents for flow cytometry and immunostaining unless specifically stated in individual method sections. These include directly conjugated primary antibodies to murine CD41-Percp-Cy5.5 (#133918), P-selectin-FITC (#304903, CD45-FITC (#103108), F4/80-PE (#123110), and Ly6G-PE (#127608), which were from BioLegend. Primary antibodies to murine CD31 (#ab119341) and F4/80 (#ab6640) were from Abcam. Secondary antibodies include goat anti-AH (#AF647, Abcam), and donkey anti-rat (#AF488, Abcam). Other reagents include Strep-Cy3 and Hoechst 33342 (#62249, Fisher Scientific), CellTracker Deep Red (#C34565 Life Technologies), and Zombie Aqua (#423101, BioLegend). *Arthrobacter ureafaciens* (#10269611001)- or *Clostridium perfringens* (#11586886001)-derived neuraminidase (sialidase) were purchased from Sigma.

### Peripheral blood cells count and platelet preparation

30 μl mouse whole blood was collected from cheek vein to a tube coated with ethylenediaminetetraacetic (EDTA). Peripheral blood cell counts and mean platelet volume were analyzed by a HemaVet 950 (Drew Scientific). Platelet isolation and preparation was performed based on our published methods [9, 32, 33], with modifications. Briefly, mouse blood was collected from the retrobulbar venous plexus via a glass capillary and added to a tube containing 3.2% (w/v) sodium citrate. Blood was then diluted 1:1 with Tyrode’s buffer (129 mM NaCl, 2.8 mM KCl, 0.8 mM MgCl_2_, 0.8 mM KH_2_PO_4_, 8.9 mM NaHCO_3_, 10 mM Hepes, 5.6 mM glucose, pH 7.4) and centrifuged at 50 g for 10 min at room temperature. The supernatant was transferred into a new tube and centrifuged at 180 g for 10 min to obtain the platelet pellet, followed by two washes with Tyrode’s buffer containing 0.5 μM prostacyclin (Sigma).

### Flow cytometry

Flow cytometry was performed following our published methods [9, 32, 33]. For lectin binding analysis, platelet staining was performed in Tyrode’s buffer with 2 μg/ml biotinylated *Maackia Amurensis* lectin II (MAL II, detecting α2,3 sialic acid, #b-1265, Vector Laboratories) and 2 μg/ml fluorescein-labeled *Ricinus Communis* agglutinin I (RCA I-FITC, recognizing desialylated galactose, #FL-1081, Vector Laboratories) for 20 min at room temperature. For biotinylated lectin, platelets were then stained with 2 μg/ml PE-streptavidin (#405203, BioLegend) for 20 min at room temperature, followed by a wash with Tyrode’s buffer. Samples incubated with PE-streptavidin only were used as negative controls. For P-selectin analysis, washed platelets stimulated with thrombin (0.3 U/ml for 5 min, Sigma) were used as positive controls. To examine platelet-neutrophil interactions, 20 μl mouse blood was collected from facial vein and added to a tube containing 3.2% sodium citrate. The cells were subsequently stained with CD41-Percp-cy5.5 and Ly6G-PE. To analyze EGFP expression on megakaryocytes and platelets from *ROSA*^*mTmG*^*;Pf4Cre* mice, peripheral blood was stained with CD41-Percp-Cy5.5 for platelet analysis. For megakaryocytes, bone marrow cells were harvested from femurs of *ROSA*^*mTmG*^*;Pf4Cre* mice and then stained with CD41-Percp-Cy5.5 and then with Hoechst33342. All samples were analyzed using a FACSCelesta (BD Bioscience).

### Quantitative reverse transcription PCR (qRT-PCR)

Total RNA was extracted from WT, *Clec4f*^+/-^, *Clec4f*^−/−^ liver tissues using Trizol (Sigma). RNA concentration was measured via Nanodrop (Thermo Fisher Scientific). Complementary DNA (cDNA) was synthesized using the M-MLV reverse transcriptase (Qiagen). Expression of *Clec4f* transcripts was analyzed using a forward primer: 5′-TCCACCTGCTTTCAGCCTTCA-3′ and a reverse primer: 5′-AGAAGACTGCCATCTGGGTCTC-3′, using a CFX96 instrument (Bio-Rad) with Fast SYBR Green Master Mix (Fisher Scientific).

### Western blot

Freshly isolated mouse livers were lysed with a lysis buffer containing 50 mM Tris pH 7.4, 150 mM NaCl, 1% Triton x-100, 1% sodium deoxycholate, 0.1% SDS, 1 mM EDTA, 0.1% SDS with protease inhibitors (1:100; Thermo Fisher Scientific). Protein samples were separated by SDS-polyacrylamide gel and transferred to a PVDF membrane. The membrane was incubated with primary goat anti-mouse CLEC4F (R&D Systems) followed by HRP-conjugated donkey anti-goat IgG (Abcam).

### Platelet desialylation, count, and survival

For in vitro desialylation of platelets, washed platelets were obtained as described above. 2 × 10^8^ platelets in Tyrode’s buffer were treated with the *Arthrobacter ureafaciens*-, or *Clostridium perfringens*-derived neuraminidase (25 mU/ml) at 37 °C for 20 min. Desialylation was confirmed by binding to RCA I-FITC and biotinylated MAL II. Platelets were labeled with 0.5 μM of CellTracker Deep Red for 20 min at 37 °C in the dark, followed by two washes with Tyrode’s buffer. Labeled platelets were then transfused into WT or *Clec4f*^−/−^ recipient mice through intravenous injections.

For in vivo desialylation, and the labeling of desialylated platelets, recipient mice were intravenously injected with neuraminidase (50 mU/mouse). Before and after neuraminidase injection, 40 μl blood was collected from the cheek vein at various time points. To determine platelet count and platelet galactose exposure in the circulation, 10 μl whole blood was diluted 1:20 in FACS buffer, then incubated with PE anti-mouse CD41 (2 μg/ml) and RCA I-FITC (2 μg/ml) for 20 min at room temperature in the dark. The percentage of platelets was determined via flow cytometry with PE anti-mouse CD41 at a low speed for 20 s. Percentage of platelet RCA I binding was defined as PE anti-mouse CD41 and RCA I-FITC positive cells per 10,000 platelets analyzed. After 120 min, recipients were sacrificed and intravascularly perfused. The liver, spleen, and other organs were then collected for imaging analysis.

### Histology, immunostaining, and microscopy

For immunofluorescent staining, tissues were fixed in 4% paraformaldehyde (PFA) at 4 °C overnight, washed in PBS, soaked in 20% sucrose at 4 °C overnight, embedded in 50% tissue freezing medium/50% OCT. Thick cryosections (20–50 μm) were used for immunostaining. In some experiments, tissues were fixed in 10% formalin for 24 h, washed in PBS, embedded in paraffin and cut into 5 μm thick sections. For immunostaining, the paraffin sections were deparaffinized, hydrated, and treated with an antigen unmasking solution (Vector) for 20 min. Sections were first blocked with 3% BSA, 3% goat serum, 3% donkey serum, and 0.3% Triton x-100 in PBS, and then stained with primary antibodies at 4 °C overnight, followed by staining with fluorescently conjugated secondary antibodies for 1 h. Antibodies used include goat anti-mouse CLEC4F (#AF2784, R&D Systems), rat anti-mouse CLEC4F (#MAB2784, R&D Systems), rat anti-mouse F4/80 (#123102, BioLegend), rabbit anti-mouse ASGR1 (# PA5-32030, Invitrogen), Alexa Fluor 488 conjugated rat anti-mouse F4/80 (#53-4801-80, Invitrogen), PE conjugated rat anti-mouse CD41 (#133906, BioLegend), and DyLight 488, Alexa Fluor 555, or DyLight 649 conjugated secondary antibodies (Jackson ImmunoResearch). Finally, sections were mounted with Fluoromount^TM^ aqueous mounting medium with DAPI (Sigma). A confocal microscope (Zeiss 710 Microscope System) was used for imaging. Volume images from the confocal image datasets were further processed with IMARIS software (Bitplane AG), and some images were presented as maximum intensity projections of the z-stacks or for three-dimensional views. The total number of bound platelets in the liver was counted using 10× or 20× magnification confocal images in a blinded manner. To determine whether platelets were associated on the surface or internalized inside of the Kupffer cell, 40× 3D z-stacks images were reconstructed and rotated to view the platelet location through various angles, and with orthogonal projection images of z-stacks.

### Desialylation, Kupffer cell isolation, and immunofluorescent staining

For desialylation of platelets in vivo, mice were injected with neuraminidase (50 mU/mouse) as described above. For specific inhibition of the macrophage galactose lectin (MGL) receptor, mice were intravenously injected with anti-MGL1/2 antibody (2 μg/g body weight, #AF4297, R&D Systems) 20 min prior to neuraminidase treatment. In some experiments, 2 × 10^8^ platelets from *ROSA*^*mTmG*^*;Pf4Cre* mice were transfused to either WT or *Clec4f*^−/−^ recipient mice, followed immediately by neuraminidase injection (50 mU/mouse). After 120 min, recipient mice were sacrificed and immediately perfused by in situ liver perfusion with 15 ml Hank’s balanced salt solution (HBSS, 1.7 ml/min, 37 °C) through the inferior vena cava, and then by 10 ml 0.1% (w/v) type IV collagenase (Worthington Biochemical Corp.) buffered in HBSS (1.7 ml/min, 37 °C). The liver was subsequently collected and transferred to a petri dish containing 10 ml cold RPMI 1640 (Hyclone) buffer. Cell suspension was prepared by gentle pipetting and then filtered through a 100 μm cell strainer on ice. Non-parenchymal cells were further separated by centrifugation for 5 min at 50 g at room temperature. The cells were then seeded onto 0.2% gelatin (Sigma) coated cover slips in RPMI 1640 supplemented with 10% fetal bovine serum (Hyclone) and 100 U/ml penicillin/streptomycin (Sigma) in a 24-well plate, and incubated for 2 h in a 5% CO_2_ atmosphere at 37 °C. Nonadherent cells were removed by gently washing with HBSS and the adherent cells were fixed with 2% PFA for 20 min at room temperature. After blocking with 1% BSA in PBS-T, the cells were incubated with primary antibodies at 4 °C overnight and then with fluorescently-conjugated secondary antibodies for 1 h. DAPI was used for counterstaining. Immunofluorescent staining was performed and the cells were analyzed using the Zeiss 710 Microscope System described above.

### Kupffer cell depletion

Mice were injected intravenously with clodronate 200 μl (#F70101C-A, FormuMax) per 20–25 g body weight 2 days before being treated with neuraminidase (50 mU/mouse). The mice were then sacrificed and perfused with PBS and 4% PFA 2 h after sialidase administration. The liver was collected for imaging analysis of Kupffer cells and platelets.

### Transmission electron microscopy (TEM)

Mouse livers were fixed by intravascular perfusion with a mixture of 2% paraformaldehyde and 2.5% glutaraldehyde in 0.1 M HCl–sodium cacodylate buffer, pH 7.2, followed up by immersion in the same fixative for 1 h. The remaining procedures followed our published methods [32, 33]. Ultrathin sections were stained with uranyl acetate and lead citrate, before being examined with a Hitachi H-7600 electron microscope equipped with a 4 megapixel digital monochrome camera and AMT-EM image acquisition software (Advanced Microscopy Techniques).

### Statistical analysis

All experiments were performed at least three times. Data are presented as mean ± SD or mean ± SEM. For parametric data, unpaired Student’s *t* test or ANOVA was used. For non-parametric data, the Mann–Whitney *U* Test was used. *P* < 0.05 was defined as statistical significance.

## Supplementary information

Supplementary Information

## Data Availability

The datasets used and/or analyzed during the current study are available from the corresponding author on reasonable request.
