# Supplementary material for: Kupffer cell receptor CLEC4F is important for the destruction of desialylated platelets in mice
Source: Cell Death Differ. 2021 May 15;28(11):3009–21. doi: 10.1038/s41418-021-00797-w (PMC8564511; doi:10.1038/s41418-021-00797-w)
Supplement: Supplementary file 1 — Supplementary Information [file 41418_2021_797_MOESM1_ESM.docx]

**Supplemental Information**

**Kupffer cell receptor CLEC4F is important for the destruction of desialylated platelets in mice**

Yizhi Jiang^1,2,3,4^*, Yaqiong Tang^1,3,4^*, Christopher Hoover^3^, Yuji Kondo^3^, Dongping Huang^2^, Damien Restagno^5^, Bojing Shao^3^, Liang Gao^3^, J. Michael McDaniel^3^, Meixiang Zhou^3^, Robert Silasi-Mansat^3^, Samuel McGee^3^, Miao Jiang^1,4^, Xia Bai^1,4,5^, Florea Lupu^3^, Changgeng Ruan^1,4,5^, Jamey D. Marth^6^, Depei Wu^1,4^, Yue Han^1,4^, Lijun Xia^3,1,4^

^1^Jiangsu Institute of Hematology, National Clinical Research Center for Hematologic Diseases, NHC Key Laboratory of Thrombosis and Hemostasis, The First Affiliated Hospital of Soochow University, Suzhou 215006, China. ^2^Department of Hematology, The First Affiliated Hospital of Wannan Medical College, Wuhu 241001, China. ^3^Cardiovascular Biology Research Program, Oklahoma Medical Research Foundation, Oklahoma City, OK 73104, USA. ^4^Collaborative Innovation Center of Hematology, Soochow University, Suzhou 215006, China. ^5^State Key Laboratory of Radiation Medicine and Protection, Soochow University, Suzhou 215123, China. ^6^Center for Nanomedicine, SBP Medical Discovery Institute, and Department of Molecular, Cellular, and Developmental Biology, University of California, Santa Barbara, CA 93106, USA.

Address correspondence to: Lijun Xia, M.D., Ph.D., Cardiovascular Biology Research Program, Oklahoma Medical Research Foundation, 825 N.E. 13^th^ Street, Oklahoma City, OK 73104. Phone: 405-271-7892. Fax: 405-271-3137. Email: [Lijun-Xia@omrf.org](mailto:Lijun-Xia@omrf.org). Or: Depei Wu (Email: [wudepei@suda.edu.cn](mailto:wudepei@suda.edu.cn)) or Yue Han (Email: hanyue@suda.edu.cn), Jiangsu Institute of Hematology, The First Affiliated Hospital of Soochow University, Suzhou 215006, China.

**Supplementary Figures**

**
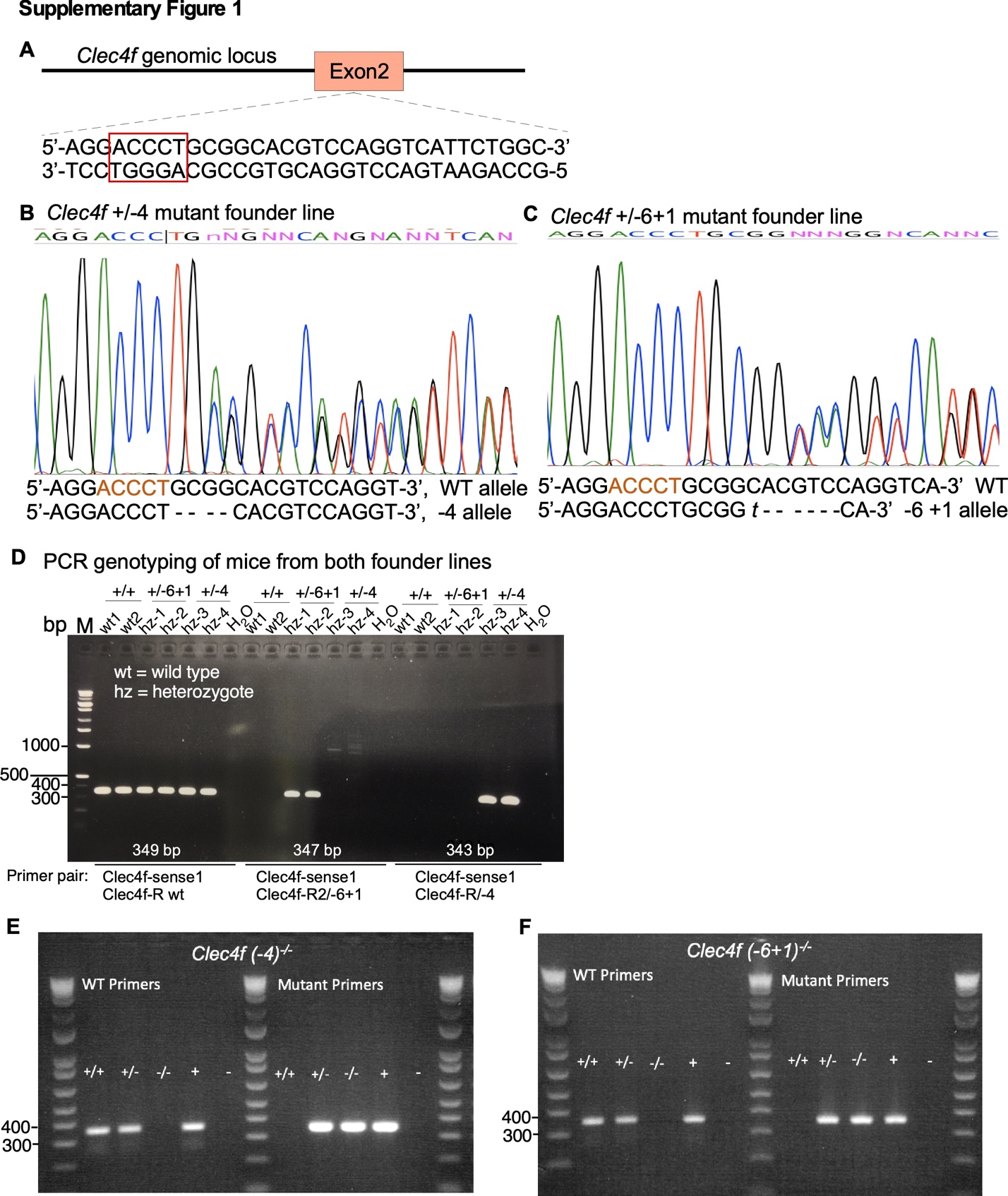
**

**Fig. S1. Generation of *Clec4f* knockout mice using CRISPR/saCas9-mediated gene editing.** **a** Schematic illustration of the *Clec4f* gene structure and sequences around the target loci. Red box: PAM. **b and c** Representative sequencing results of the F0 founder with bi-allelic frameshifting indel-mutations of *Clec4f*. One identified indel-mutation is named as *Clec4f* -4, in which -4 indicates a 4-bp deletion (b). Another is termed as *Clec4f* -6+1, in which the -6 indicates a 6-bp deletion with an insertion of a *t* (+1) (c). The hyphens indicate deletion nucleotides. Red letters of the WT allele indicate PAM. **d, e,** and **f** PCR genotyping results of the founder line, WT littermates, *Clec4f* -4 and *Clec4f* -6+1 heterozygotes, and *Clec4f* -4 and *Clec4f* -6+1 homozygotes.

.

**
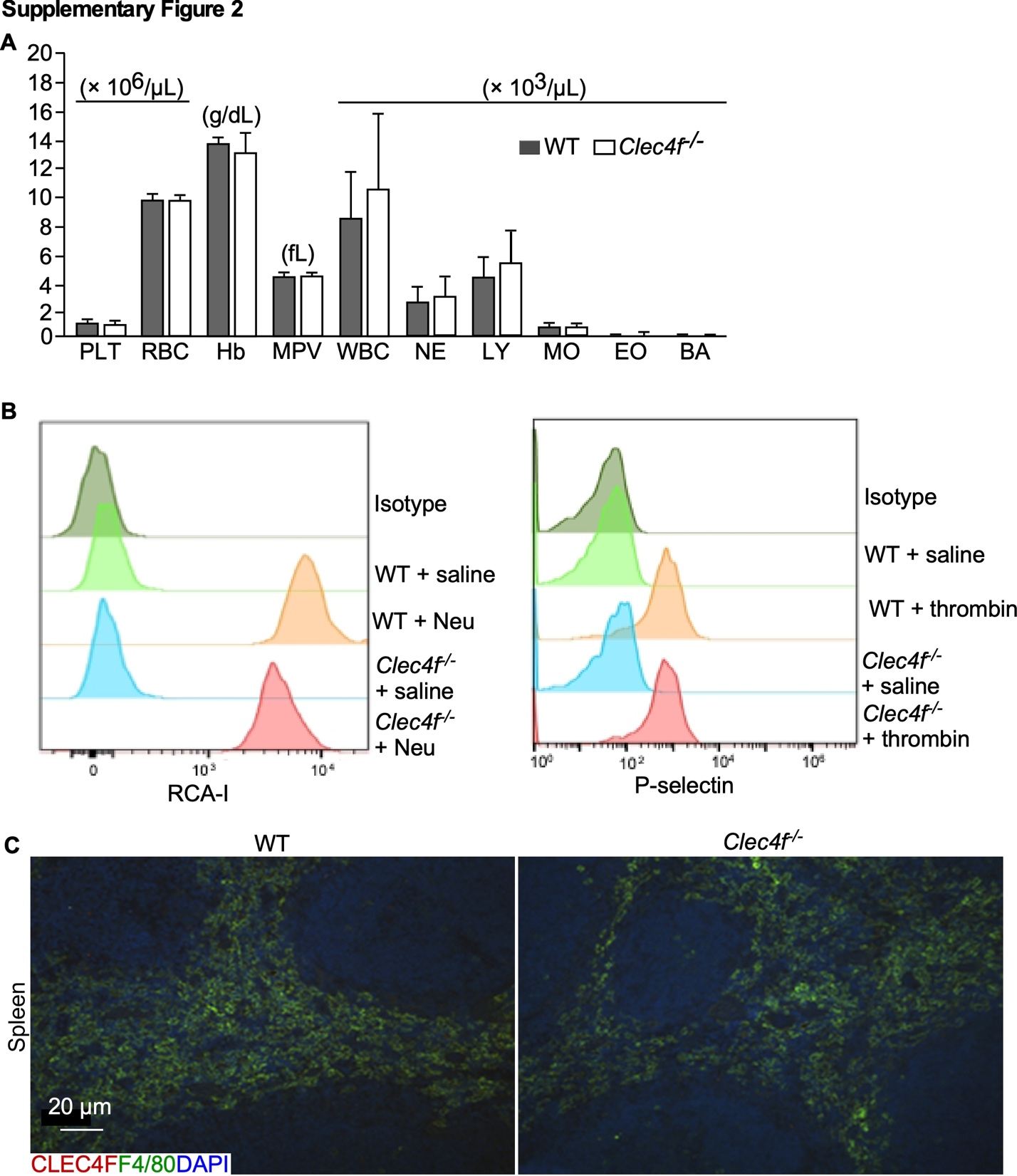
Fig. S2. Characterization of *Clec4f* knockout mice.** **a** Analysis of peripheral blood cells count in WT and *Clec4f^-/-^* mice. PLT, platelet; RBC, red blood cell; Hb, hemoglobin; MPV, mean platelet volume; WBC, white blood cell; NE, neutrophil; LY, lymphocyte; MO, monocyte; EO, eosinophil; BA, basophil. Means ± SD, n = 7 mice per genotype. **b** Flow cytometry results of the platelet sialylation status and response to agonist thrombin. RCA-1 detects desialylation. Surface expression of P-selectin marks activation of platelets. **c** Representative images of immunofluorescent staining of formalin-fixed paraffin-embedded spleen sections. Kupffer cell (CLEC4F, red or F4/80, blue). DAPI, blue. Results are representative of three separate experiments.

**
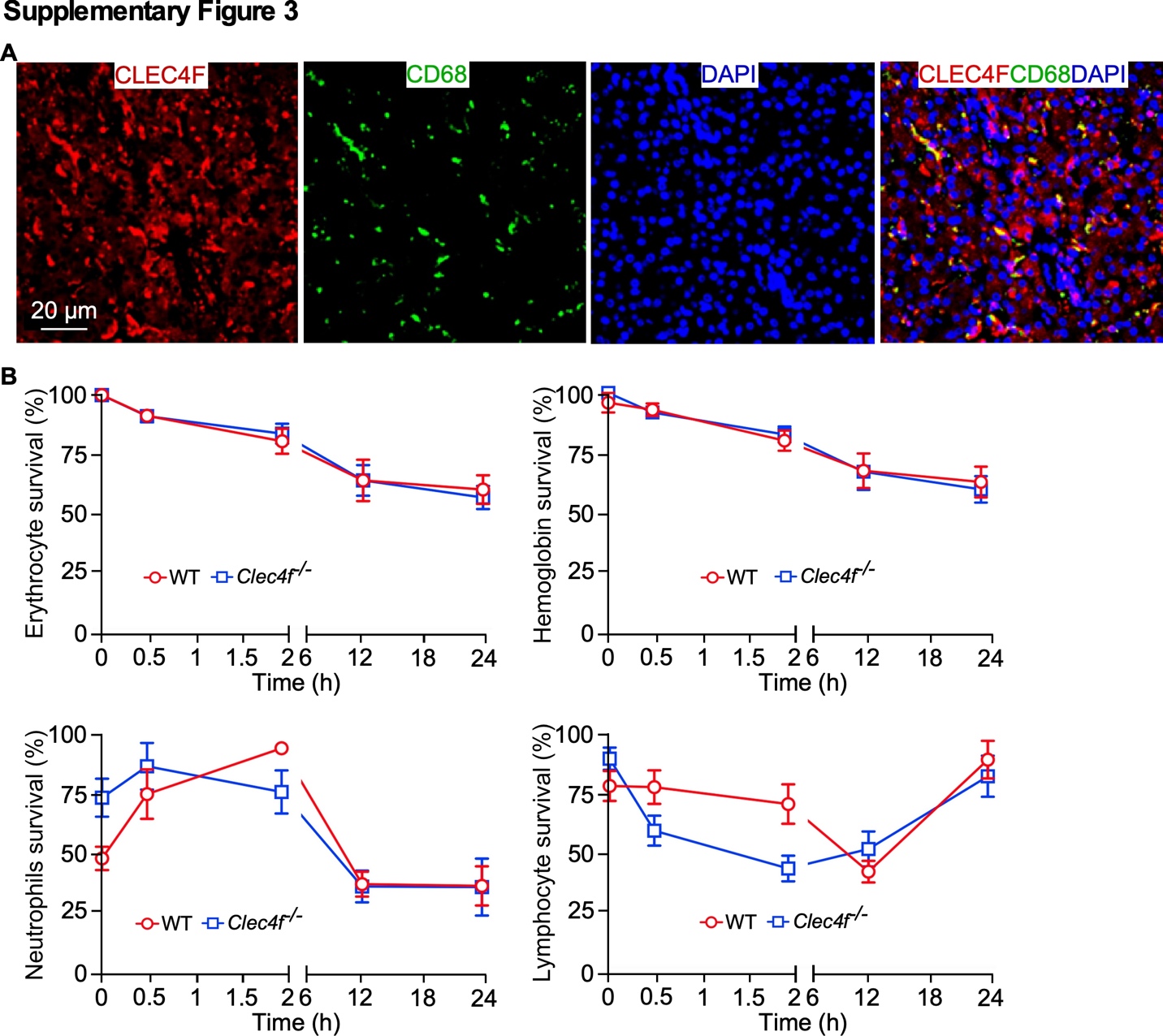
**

**Fig. S3. a** Representative confocal images of de-identified formalin-fixed paraffin-embedded human liver sections. Yellow color in the right panel indicates colocalized CLEC4F with CD68-positive Kupffer cells. **b** Peripheral blood cell values of WT and *Clec4f^-/-^* mice were analyzed using a Hemavet Hematology Analyzer. Means ± SD, n = 7 mice per genotype.


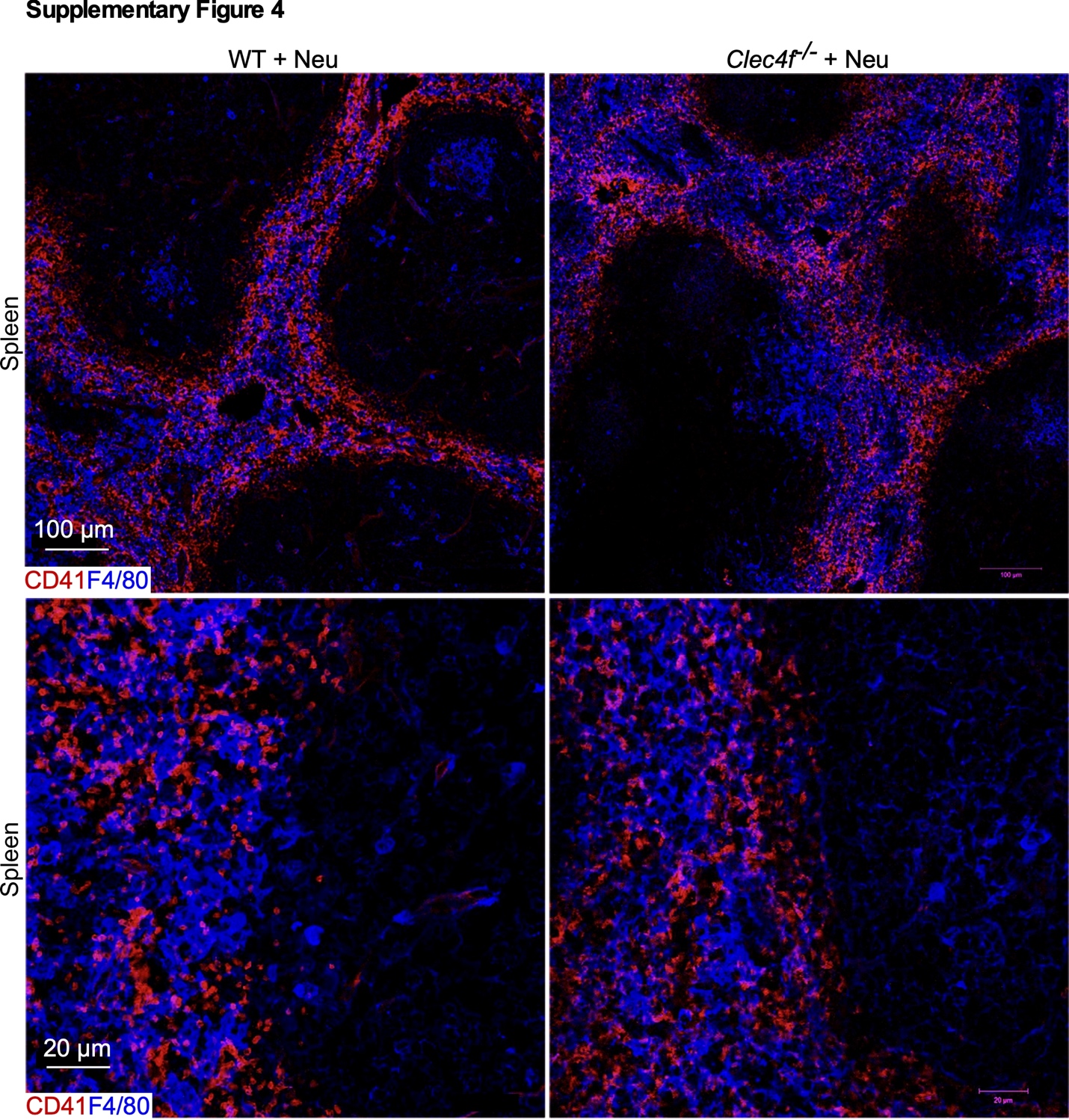


**Fig. S4. There is no obvious difference of platelet association with macrophages in the spleen of WT and *Clec4f*^-/-^ mice after neuraminidase treatment**. Representative immunofluorescent images of platelets (CD41, red) with macrophages (F4/80, blue) in the spleen sections.

**
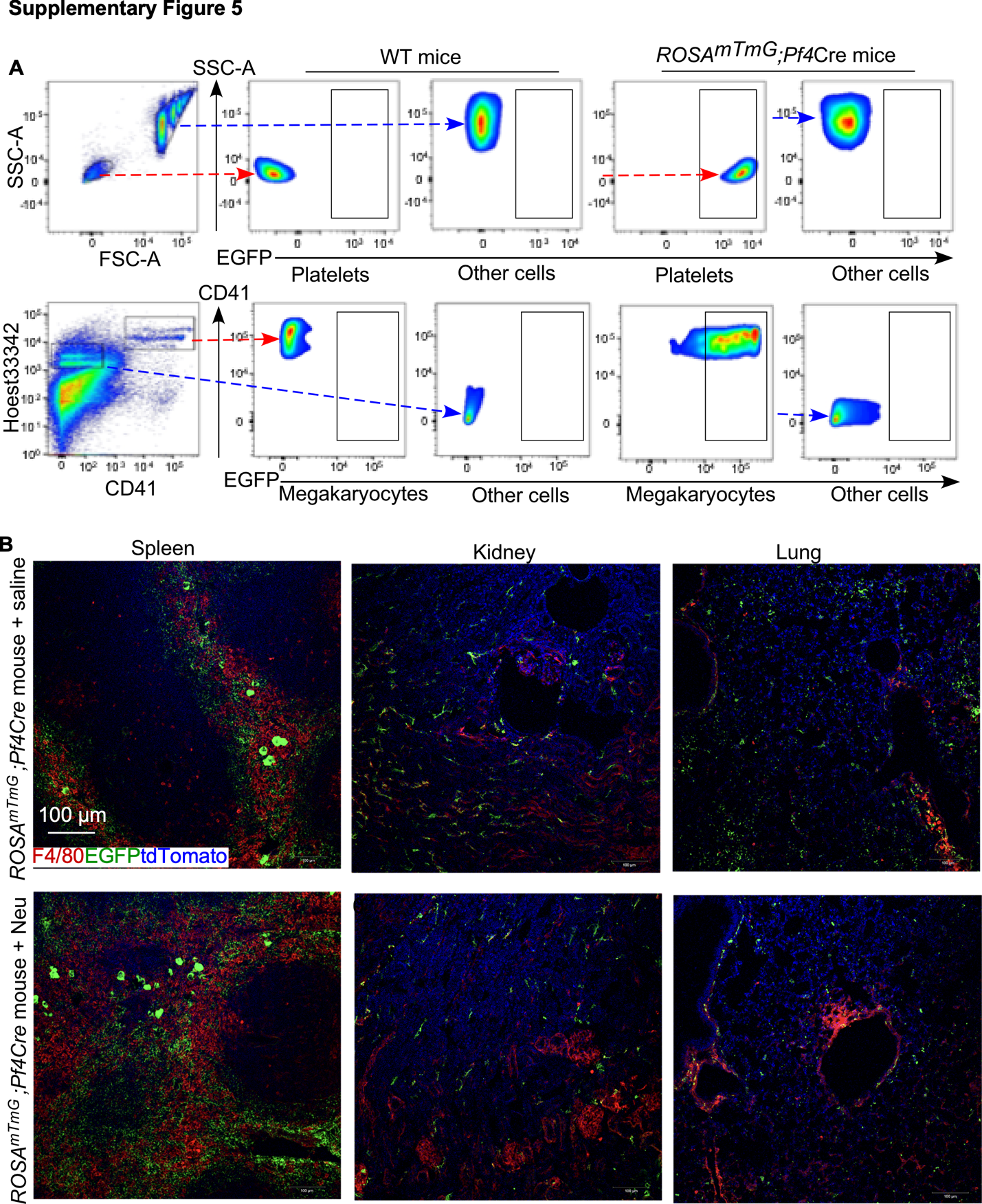
**

**Fig. S5. Characterization of *ROSA^mTmG^;Pf4Cre* mice.** **a** Flow cytometry results show that bone marrow megakaryocytes and peripheral platelets from *ROSA^mTmG^;Pf4Cre* mice specifically express EGFP. Platelets were identified based on their SSC and FSC properties. Megakaryocytes were identified based on their polyploid nuclei marked by Hoechst33342 and expression of CD41. Red arrows indicate EGFP-positive megakaryocytes and platelets. Blue arrows mark other cells. **b** Representative immunofluorescent images showing association of platelets (EGFP, green) with macrophages (F4/80, red) in the spleen, kidney, and lung of *ROSA^mTmG^;Pf4Cre* mice 2 h after neuraminidase treatment. DAPI, blue. tdTomato (other cell types except platelets). Control, saline treatment. Neu, neuraminidase.

**
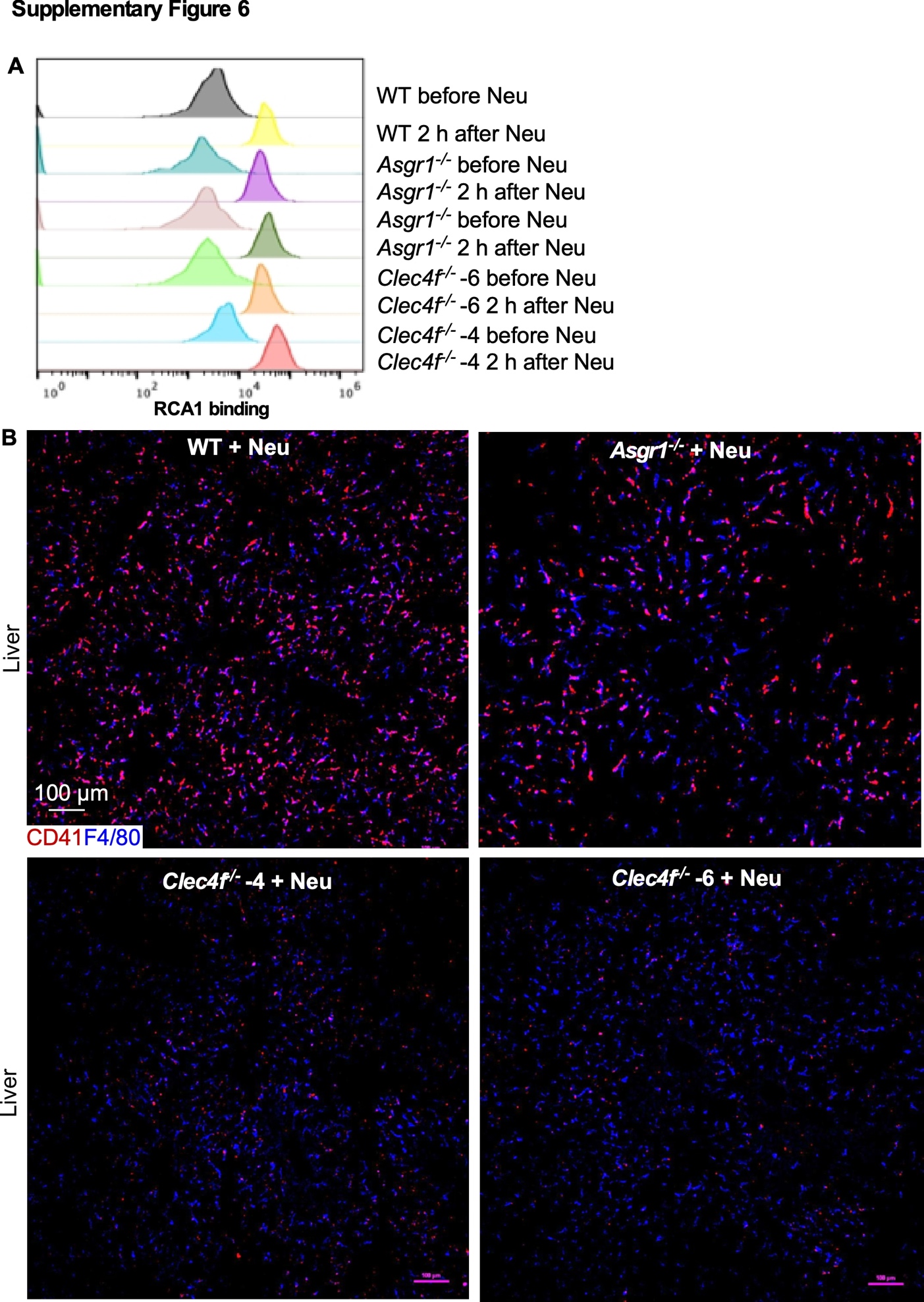
**

**Fig. S6. CLEC4F but not ASGR1 is a primary receptor mediating desialylated platelet clearance in mice treated with** ***Clostridium perfingens*-derived neuraminidase**. **a** Flow cytometric analysis of RCA1 profiles of different mouse mutants and WT controls before and 2 h after injections of *Clostridium perfringens*-derived neuraminidase. **b** Representative confocal images of liver cryosections of mice treated with *Clostridium perfringens*-derived neuraminidase.

**
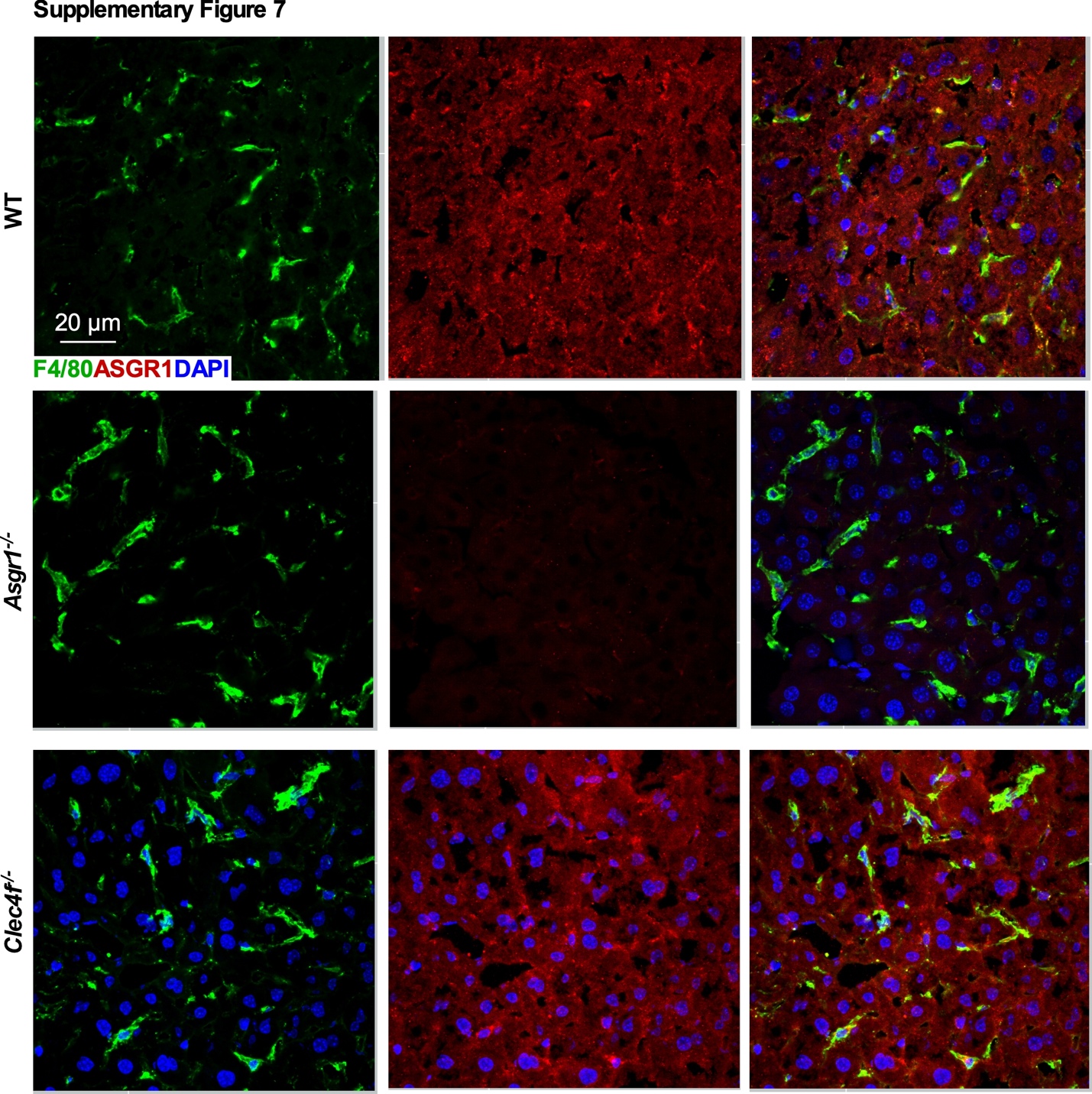
**

**Fig. S7. The expression of ASGR1 is not altered in *Clec4f^-/-^* mice**. Representative confocal images of liver cryosections of different mutant mice. *Asgr1^-/-^* liver was used a negative control.

**
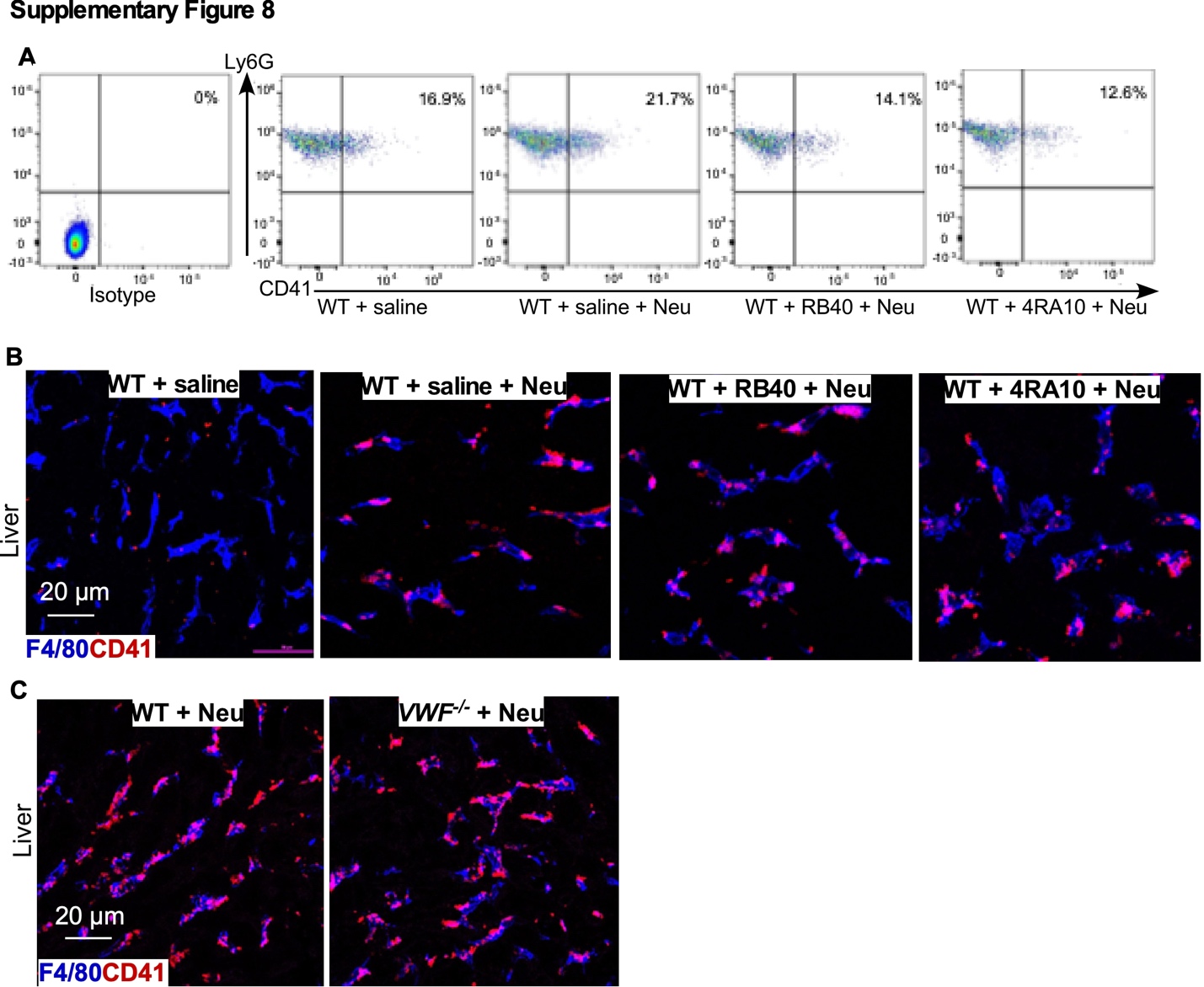
**

**Fig. S8. Platelet P-selectin-mediated platelets/leukocyte complex or VWF-mediated adhesion of platelets to Kupffer cells does not contribute to desialylated platelet clearance by the Kupffer cells. a** Flow cytometry analysis of platelet P-selectin-mediated platelets/leukocyte complex formation with or without the presence of RB40 or 4RA10. The analysis was performed on blood from WT mice that were injected with RB40 or 4RA10 (200 ug/mouse) followed by neuraminidase treatment. RB40, a functional blocking antibody against murine P-selectin. 4RA10, a functional blocking antibody against murine P-selectin glycoprotein ligand 1 (PSGL-1. Ly6G marks granulocytes including neutrophils. **b** Representative confocal images of liver cryosections of mice from different experimental groups. **c** Representative confocal images of liver cryosections of WT and VWF-deficient mice treated with neuraminidase.
